# A multiband circular polarization selective metasurface for microwave applications

**DOI:** 10.1038/s41598-021-81435-w

**Published:** 2021-01-19

**Authors:** Syed Muhammad Qasim Ali Shah, Nosherwan Shoaib, Fahad Ahmed, Akram Alomainy, Abdul Quddious, Symeon Nikolaou, Muhammad Ali Imran, Qammer H. Abbasi

**Affiliations:** 1 Research Institute for Microwave and Millimeter-Wave Studies (RIMMS), National University of Sciences and Technology (NUST), Islamabad, 44000 Pakistan; 2 School of Electronic Engineering and Computer Science, Queen Mary University of London, London, E1 4NS UK; 3 KIOS Research and Innovation Center of Excellence, University of Cyprus, 2109 Nicosia, Cyprus; 4 Frederick Research Center (FRC) and Department of Electrical Engineering, Frederick University, 1036 Nicosia, Cyprus; 5 James Watt School of Engineering, University of Glasgow, Glasgow, G12 8QQ UK

**Keywords:** Metamaterials, Metamaterials, Electrical and electronic engineering

## Abstract

In this research article, a multiband circular polarization selective (CPS) metasurface is presented. A reciprocal bi-layered metasurface is designed by introducing the chirality in the structure. The top layer of the proposed metasurface is composed of circular split-ring resonator with a cross shape structure inside it. The same structure is printed on the bottom side of the proposed metasurface by rotating it at an angle of 90° to achieve chirality in the structure. The proposed metasurface is able to add CPS surface capability between 5.18 and 5.23 GHz for y-polarized incident wave. For the frequency band of 5.18–5.23 GHz, the transmission goes up to − 4 dB, while the polarization extinction ratio (PER) reaches up to − 27.4 dB at 5.2 GHz. Similarly, for x-polarized incident wave, three strategic CPS operating bands are achieved within the frequency ranges of 10.64–10.82 GHz, 12.25–12.47 GHz, and 14.42–14.67 GHz. The maximum PER of 47.16 dB has been achieved for the 14.42–14.67 GHz frequency band at 14.53 GHz. Furthermore, the response of the metasurface does not vary against oblique incidences up to 45°. The simple structure, angular stability, multiband and miniaturized size make this metasurface an outstanding applicant for polarization conversion and biomedical applications.

## Introduction

Metamaterials exhibit extraordinary properties and provide ample opportunity to control and manipulate the polarization, amplitude and phase of an electromagnetic wave. Chiral metamaterials, a special subclass of metamaterials, composed of a structure which lack mirror symmetry and its mirror image cannot be superimposed. Chirality is a significant characteristic of materials in the organic world, which exits in natural molecules like proteins, amino acids and carbohydrates^1^. Chiral metamaterial received significant attention since the Pendry et al.^2^ introduced the chirality to achieve negative refractive index^3^ (NRI). Although, metamaterials have many advantages, howerver, these are replaced by metasurfaces^4^ (i.e., 2D analog of metamaterial). The reason being 3D metamaterials face significant fabrication complexities, have bulky size and inherently have a very narrow bandwidth. Owing to the chirality in metasurfaces, a wide variety of remarkable electromagnetic properties can be realized such as giant gyrotropy^5,6^, optical activity (90°-polatization rotation^7–10^), circular dichroism^11,12^, polarization selectivity^13–15^ and asymmetric transmission^16–20^.

The asymmetric transmission is realized by Fedotov^21^ in 2006 and it has now become a focal point of research to achieve asymmetric linear-to-linear polarization (optical activity) and circular polarization selectivity. Various techniques have been reported on the asymmetric linear-to-linear polarization area through the use of bi-layered^22,23^ and multi-layered^24,25^ chiral structures. Currently, scientific community has directed attention towards realizing asymmetric linear-to-circular polarization^26–32^. As it is a challenging task to achieve asymmetric linear-to-circular (CPS) like the ones in^27,28,32^, however, multi-layered structures can be deployed where multi-band or enhanced bandwidth is required.

The multiband circular polarization selectivity was achieved using multi-layered metallic structures on substrate sheet when they only operated at normal incidence^27,28^. The multi-layered structures cause high transmission loss, while reflection coefficient increase due to poor wave impedance matching^32^. Therefore, the research trend is to achieve circular polarization selectivity by using low cost bi-layered chiral metasurfaces. The dual band circular polarization selective metasurfaces using bi-layered structure has been demonstrated^29–32^. Zhang et al. achieved circular polarization selectivity at two bands by using bi-layered inverted G-shaped structure^31^.

Khan et al. proposed a highly efficient chiral metasurface^32^ in which CPS operation has been achieved only at the resonance frequency of 14.79 GHz with transmission magnitude of − 4.5 dB, however, the design worked only for normal incidence. From the aforementioned literature, it can be realized that the designs are either low efficient, operate only at the resonance frequencies with narrow bandwidth, or work at normal incidence. In this context, achieving high efficient multiband circular polarization selectivity along with the angular stability can be attractive due to its capability to integrate with various practical applications in polarization manipulation and biomedical devices^33,34^.

In this paper, a novel and efficient bi-layered metasurface is presented with multiband CPS characteristics. The circular polarization selectivity is achieved within the frequency range of 5.18–5.23 GHz for y-polarization. While for x-polarization, the CPS operation is achieved in three frequency bands, i.e., 10.64–10.82 GHz, 12.25–12.47 GHz and 14.42–14.67 GHz. For C-band (5.18–5.23 GHz), transmission goes upto -4 dB which is the highest magnitude that has been achieved till to date to the best of authors’ knowledge. Moreover, the metasurface operates very well for both x-polarizaton (TM) and y-polarization (TE) against oblique incidence up to 45°, offering assistance in polarization conversion applications.

## Theoretical analysis

Let us consider that a plane wave is travelling along − z direction whose incident electric field (*E*_*i*_) and transmitted electric field (*E*_*t*_) can be stated as^35^:

1 $${E}_{i}(x,y,z,t)=\left[\begin{array}{c}{E}_{xi}\\ {E}_{yi}\end{array}\right]{e}^{-i(kz-wt)}$$

2 $${E}_{t}(x,y,z,t)=\left[\begin{array}{c}{E}_{xt}\\ {E}_{yt}\end{array}\right]{e}^{-i(kz-wt)}$$

where *w* and *k* represent the frequency and wave number, respectively, while the complex amplitudes (*E*_*x*_ and *E*_*y*_) represent the x-and y-components of electric field. The transmission matrix (T-matrix) for linear polarization can be defined as^36^:

3 $$\left[\begin{array}{c}{E}_{xt}\\ {E}_{yt}\end{array}\right]=\left[\begin{array}{cc}{T}_{xx}& {T}_{xy}\\ {T}_{yx}& {T}_{yy}\end{array}\right]\left[\begin{array}{c}{E}_{xi}\\ {E}_{yi}\end{array}\right]={T.C}_{Linear}\left[\begin{array}{c}{E}_{xi}\\ {E}_{yi}\end{array}\right]$$

Here *T*_*xx*_ and *T*_*xy*_ represent the co- and cross-component of transmission for x-polarized wave, respectively. While *T*_*yx*_ and *T*_*yy*_ indicates the cross- and co-component of transmission for y-polarized wave, respectively. The transmission component (T.C) can be written as *T.C* = *E*_*t*_*/E*_*i*_. Moreover, the transmission matrix (T-matrix) for circular polarization can be expressed as^36^:

4 $$\left[\begin{array}{c}{E}_{+t}\\ {E}_{-t}\end{array}\right]=\left[\begin{array}{cc}{T}_{+x}& {T}_{+y}\\ {T}_{-x}& {T}_{-y}\end{array}\right]\left[\begin{array}{c}{E}_{xi}\\ {E}_{yi}\end{array}\right]={T.C}_{cir}\left[\begin{array}{c}{E}_{xi}\\ {E}_{yi}\end{array}\right]$$

Here + and − represent the right-hand circular polarization (RHCP) and left-hand circular polarization (LHCP), respectively. The *T.C*_*cir*_ matrix can be transformed to the Cartesian basis such as^27,32^:

5 $${T}_{cir}=\left[\begin{array}{cc}{T}_{+x}& {T}_{+y}\\ {T}_{-x}& {T}_{-y}\end{array}\right]=\frac{1}{\sqrt{2}}\left[\begin{array}{cc}{T}_{yx}-i{T}_{xx}& {T}_{yy}+i{T}_{xy}\\ {T}_{xx}-i{T}_{yx}& {T}_{yy}-i{T}_{xy}\end{array}\right]$$

## Simulated results

### Design of CPS metasurface

**Figure 1:**
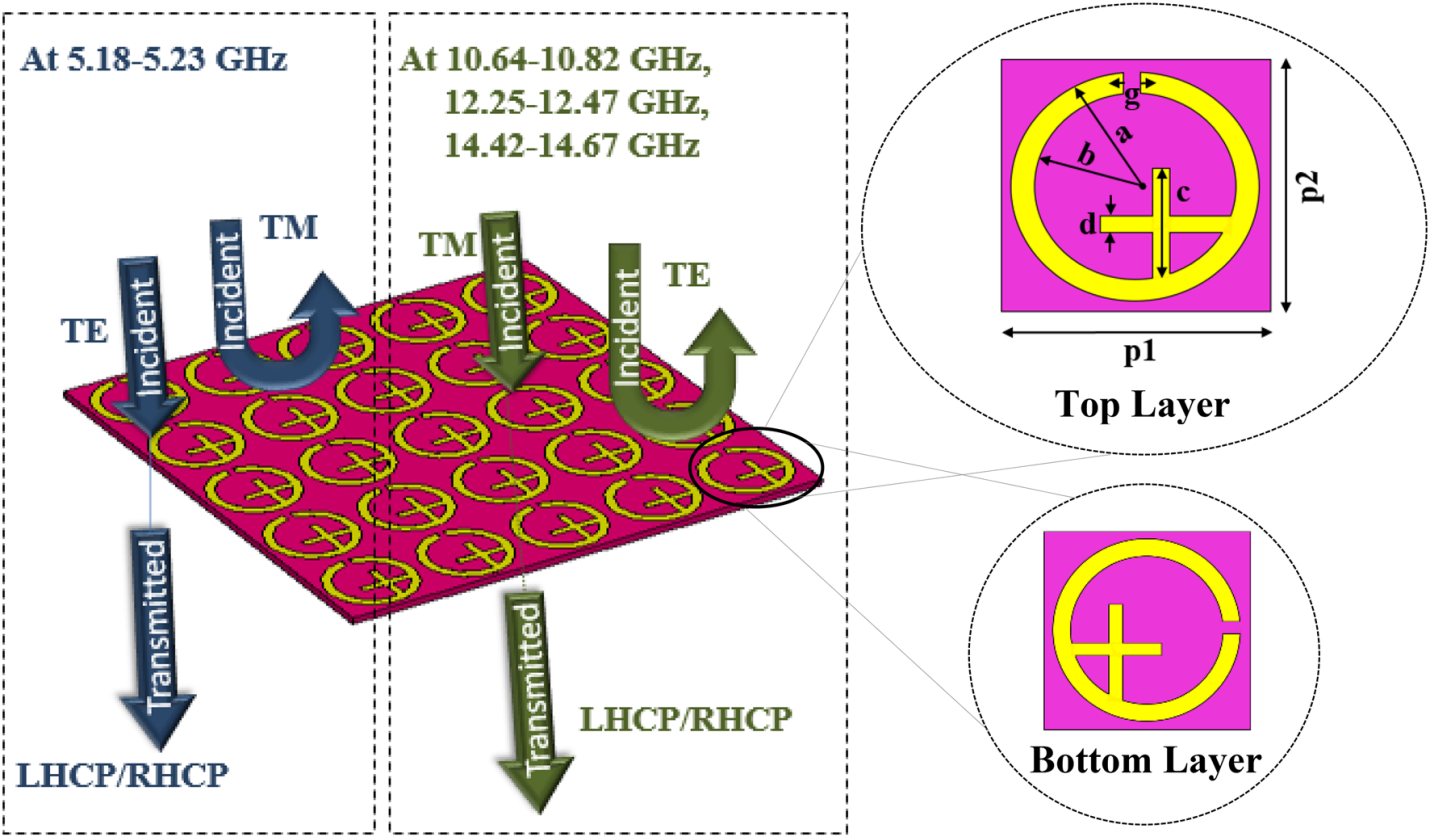
Schematic diagram of the circular polarization selective metasurface along with geometric configuration of the top and bottom metallic layers of the unit cell.

The schematic view of the proposed circular polarization selective metasurface along with the top and bottom layers of the unit cell are presented in Fig. 1. The designed metasurface is printed on both sides of the Rogers RT5870 substrate (*ɛ*_*r*_ = 2.33, loss tangent = 0.0012) having thickness of 1.57 mm. The 17 µm copper cladding (with a conductivity of 5.80 × 10^7^ S/m) is employed to design the metallic split-ring resonator (SRR) and cross-element resonator. The design on the back side of the substrate is rotated by an angle of 90° compared to the front side of the substrate to achieve chirality in the structure. The unit cell is repeated itself across the x–y plane with the same period of 10 mm to generate an array. To optimize the proposed design, its dimensions are varied with the intention to obtain desired results. The optimized parameters of the unit cell are as follows: p1 = 10, p2 = 10, a = 4.5, b = 3.55, d = 0.75, c = 5 and g = 0.75 (all units in mm).

**Figure 2:**
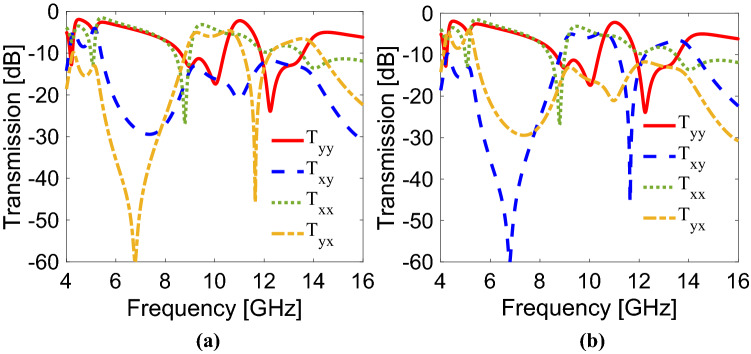
Magnitude of co- and cross-transmission components in (**a**) backward direction, (**b**) forward direction.

To simulate and analyze the proposed metasurface, a full electromagnetic numerical solver, CST Studio Suite is utilized. To simulate the metasurface, periodic and open boundary conditions along x- and y-directions along with Floquet ports along z-direction are used. The simulated transmission components (i.e., *T*_*yy*_,* T*_*xy*_,* T*_*yx*_,* T*_*xx*_) for both transverse-electric (TE) and transverse-magnetic (TM) incident waves propagating along the backward (−z) and forward (+z) directions are presented in Fig. 2a,b, respectively. For y-polarization, a CPS operating band is achieved within the frequency band of 5.18–5.23 GHz while three frequency bands of the circular polarization selectivity ranging from 10.64 to 10.82 GHz, 12.25 to 12.47 GHz and 14.42 to 14.67 GHz are realized for x-polarization in the backward direction as displayed in Fig. 2a. On the other hand, it can be manifested from Fig. 2b that no CPS operating band is achieved over these frequency bands: 5.18–5.23 GHz, 10.64–10.82 GHz, 12.25–12.47 GHz and 14.42–14.67 GHz when either x-polarization (TM) or y-polarization (TE) is incident from the forward direction. For instance, from Fig. 2a, it can be realized that the co- (*T*_*yy*_) and cross- (*T*_*xy*_) components of the TE wave are approximately equal with transmission magnitude of about 0.63 (− 4.0 dB) at 5.2 GHz while in Fig. 2b, transmission components (*T*_*yy*_ and *T*_*xy*_) are not equal at 5.2 GHz, leading to no CPS operation in the forward direction. Similarly, in the case of TM wave, transmission components (*T*_*xx*_ and *T*_*yx*_) are not equal over three claimed bands in the forward direction.

**Figure 3:**
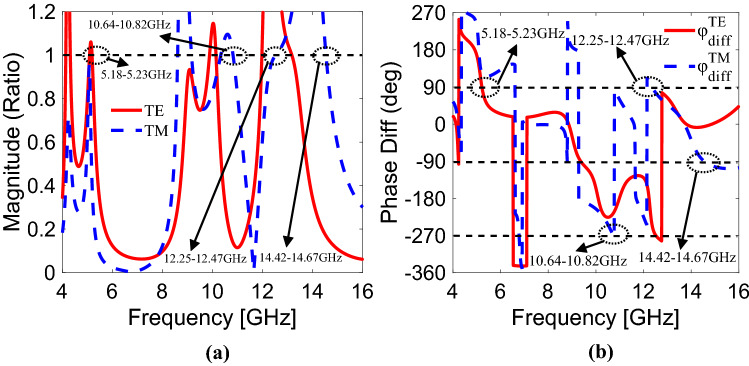
For both y-polarization and x-polarization, (**a**) magnitude ratio, (**b**) phase difference between co- and cross-components.

The CPS operating bands are achieved when magnitude ratio and phase difference between co- and cross-components lie within (0.85–1.15) and (85°–95°), respectively. In Fig. 3a, the magnitude ratio^37^ (*T*_*xy*_*/T*_*yy*_) remains within (1 ± 0.15) while the phase difference^37^ (*φ*_*diff*_ = *φ*_*xy*_* − φ*_*yy*_) between 5.18 and 5.23 GHz remains nearly 90° as indicated in Fig. 3b. Therefore, pure circular polarization selectivity is achieved within 5.18–5.23 GHz when y-polarization is incident. Similarly, when x-polarization is incident on the metasurface, the magnitude ratio (*T*_*yx*_*/T*_*xx*_) remains nearly equal to 1 and their phase difference (*φ*_*diff*_ = *φ*_*yx*_* − φ*_*xx*_) remains around ± 90° or odd multiples of ± 90° over the frequency ranges of 10.64–10.82 GHz, 12.25–12.47 GHz and 14.42–14.67 GHz. From this, it can be stated that the pure circular polarization selectivity is achieved at all aforementioned frequency bands when TM wave is incident.

### Circular polarization transmission

**Figure 4:**
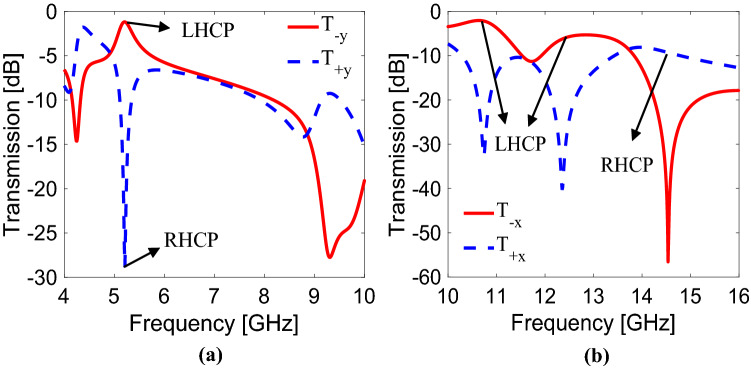
Circular polarization transmission components for (**a**) y-polarization, (**b**) x-polarization.

The transmission components for circular polarization (*T.C*_*cir*_) can be obtained by using *T.C*_*Linear*_ components. *T.C*_*cir*_ can be calculated for both x- and y-polarizations by using Eq. (5). From Fig. 4a, for y-polarized wave, the circular polarization transmission for LHCP has a maximum value of − 1.18 dB while for RHCP, it has a minimum value of − 28.7 dB at 5.21 GHz. There is a remarkable difference between the values of LHCP and RHCP at 5.21 GHz such that LHCP is permitted while RHCP is restricted to pass through the metasurface at 5.21 GHz. This indicates that the incident y-polarization is converted to the pure LHCP at 5.21 GHz. Similarly, for x-polarized wave, the circular polarization transmission for LHCP reaches up to values of − 2.17 dB at 10.74 GHz, − 6.23 dB at 12.35 GHz and − 56.5 dB at 14.53 GHz, respectively, as shown in Fig. 4b. On the other hand, circular polarization transmission for RHCP has minimum values of − 32.6 dB at 10.74 GHz, − 40.19 dB at 12.35 GHz and has a value of − 9.29 dB at 14.53 GHz. It shows that a pure LHCP is obtained at 10.74 GHz and 12.35 GHz while a pure RHCP is achieved at 14.53 GHz when x-polarization is incident.

### Polarization extinction ratio

Polarization extinction ratio (PER) is another criterion to demonstrate the functionality of polarization conversion. It is a parameter that verifies the efficiency of circular polarization selectivity. For y-polarization, PER can be calculated by using Eq. (6)^37^.

6 $$PER=20*{log}_{10}\frac{{|T}_{+y}|}{{|T}_{-y}|}$$

**Figure 5:**
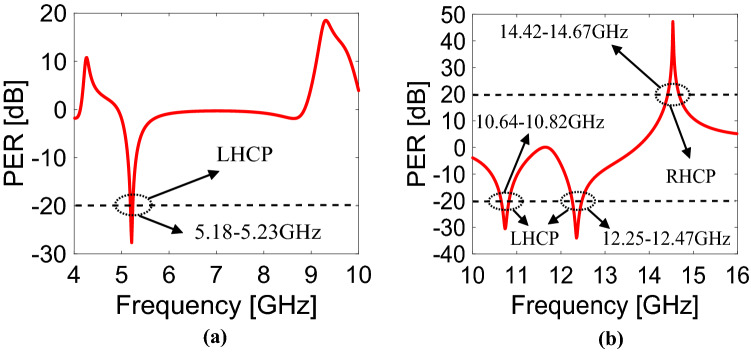
Polarization extinction ratio for (**a**) y-polarization, (**b**) x-polarization.

To find PER for x-polarization, *T*_+*y*_ and *T*_*−y*_ need to be replace with *T*_+*x*_ and *T*_*−x*_, respectively. For pure CPS operation, the graph of PER should be greater than + 20 dB or less than − 20 dB. In Fig. 5a for y-polarization, PER remains below − 20 dB in the frequency band of 5.18–5.23 GHz with the amplitude of PER goes up to − 27.4 dB at 5.21 GHz. This explains that the TE wave is converted to LHCP in the frequency band of 5.18–5.23 GHz. Similarly, in Fig. 5b for x-polarization, PER remains below − 20 dB in the frequency bands of 10.64–10.82 GHz and 12.25–12.47 GHz while it remains above + 20 dB in the frequency band of 14.42–14.67 GHz. The amplitudes of PER reaches up to − 30.4 dB and − 33.9 dB at 10.74 GHz, 12.35 GHz, respectively, while at 14.53 GHz, the amplitude of PER goes up to 47.16 dB which is the highest amplitude of PER that has been achieved till to date to the best of authors’ knowledge. This indicates that the TM wave is converted to LHCP in the frequency bands of 10.64–10.82 GHz and 12.25–12.47 GHz and RHCP in the frequency band of 14.42–14.67 GHz.

### Angular stability

**Figure 6:**
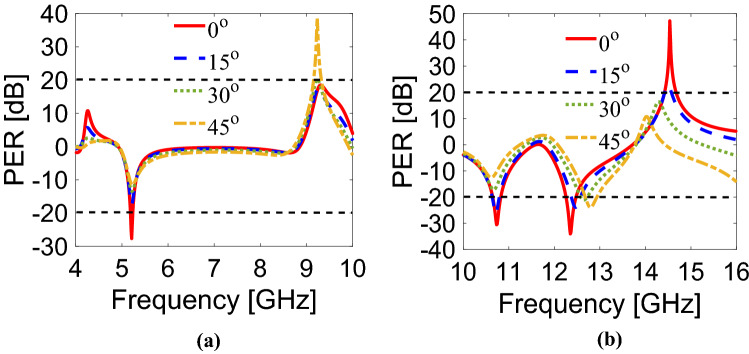
Polarization extinction ratio at different angles for (**a**) y-polarization, (**b**) x-polarization.

The behavior of the CPS metasurface is analyzed at different incidence angles because of the stability requirement for many applications. It can be seen from Fig. 6a that the proposed structure is angularly stable up to an oblique incidence of 45° for y-polarization. Similarly, for x-polarization, the metasurface remains stable up to 45° for the first band (10.64–10.82 GHz) while for the second (12.25–12.47 GHz) and third band (14.42–14.67 GHz), it remains stable up to 15° as presented in Fig. 6b. The response of the designed metasurface is changed at 30° and 45° for the two bands (12.25–12.47 GHz, 14.42–14.67 GHz) because of the larger electrical size of the unit cell. Moreover, it is interesting to see that for y-polarization, there is an additional band at resonant frequency of 9.23 GHz which is also performing CPS operation as shown in Fig. 6a.

### Surface currents analysis

**Figure 7:**
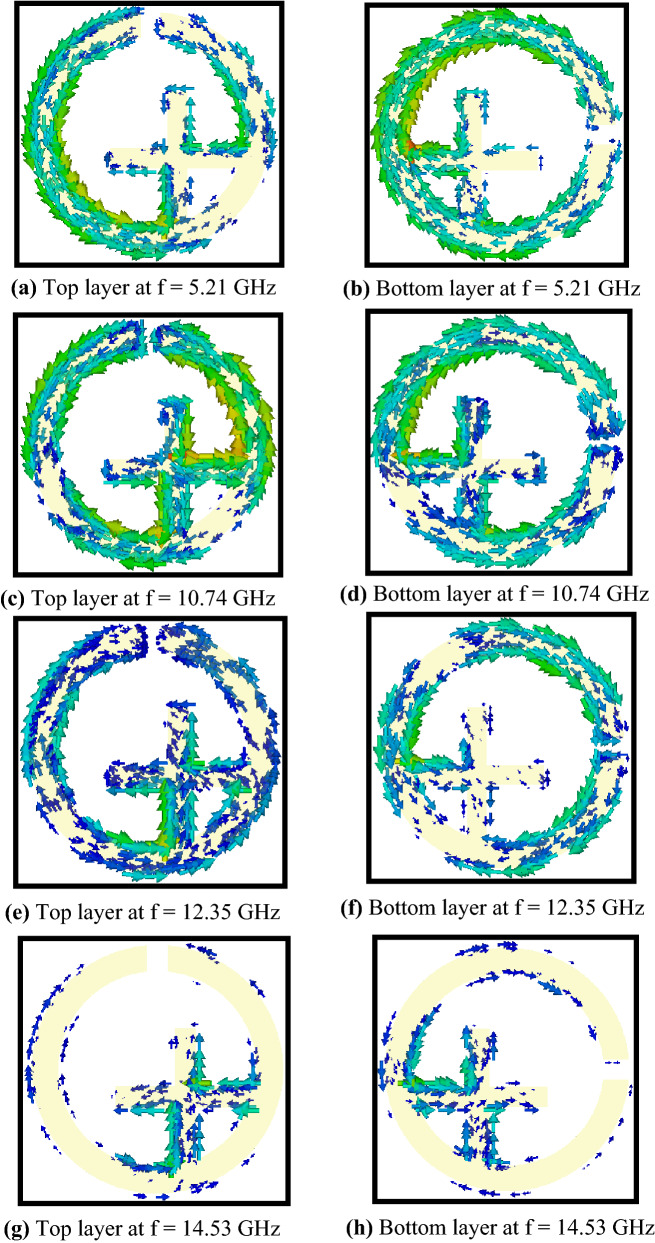
Surface current distributions on the top and bottom layers of the proposed metasurface at different frequencies.

The physical behaviour behind the circular polarization selectivity can be explained by analysing the surface currents. The circular polarization conversion is the outcome of interlayer (transverse) magnetic dipole to magnetic dipole coupling at the resonances. The electromagnetic waves with in the metallic layers can be shown by eigenmodes of the resonators^28,32^. Figure 7 describes the induced surface currents at multiple frequencies, in which (a), (b) correspond for incident TE wave, while (c)–(h) correspond for incident TM wave. From Fig. 7a,b, it can be manifested that the surface currents on the top and bottom layers of the SRR design at 5.21 GHz are in the opposite (antiparallel) direction which show antiparallel magnetic dipole coupling^32^ along the external electric field. Moreover, the surface current is in parallel direction on one metallic strip while it is in antiparallel direction on the other metallic strip of the bottom layer with respect to the top layer. Thus creating the cross coupling between the electric and magnetic fields in the bi-layered chiral metasurface^28^. The current directions on the top and bottom layers of the structure evaluate the type of the transmitted wave (i.e., RHCP or LHCP). Therefore, at 5.21 GHz, the antiparallel currents on the two layers depicts that the transmitted wave is LHCP. Furthermore, it is apparent from Fig. 7c,d that the antiparallel and parallel surface currents exist on the SRR and metallic strips of the bottom layer with respect to the top layer respectively, leading to an LHCP wave at 10.74 GHz for incident TM wave. Similarly, Fig. 7e,f show that the directions of surface currents on the structure represent that LHCP wave is transmitted at 12.35 GHz for TM wave. In addition, from Fig. 7g,h, for incident TM wave at 14.53 GHz, the surface currents on SRR structure and both metallic strips are in same (parallel) direction which show a parallel magnetic dipole coupling^32^ along the external electric field. Therefore, it can be stated that the transmitted wave is RHCP at 14.53 GHz.

## Experimental results and discussion

**Figure 8:**
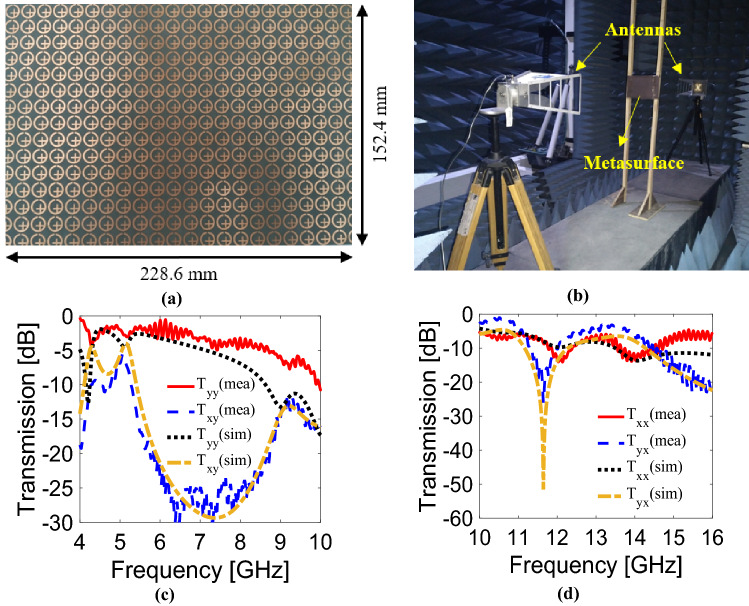
(**a**) Photograph of the fabricated metasurface, (**b**) experimental measurements setup, (**c**) comparison between simulated and measured results for TE wave, (**d**) comparison between simulated and measured results for TM wave.

In order to validate the simulated results, the proposed CPS metasurface was fabricated on Rogers 5870 substrate. The fabricated sample (having cross-section of 228.6 × 152.4 mm^2^) consists of 22 × 15 unit cells, as shown in Fig. 8a. The measurements were carried out in an anechoic chamber while the Rogers sheet was placed between two horn antennas as presented in Fig. 8b. These antennas were attached to the Anritsu-MS46122B (vector network analyzer) via coaxial cables. In order to achieve co-polarized transmission (*T*_*yy*_ or *T*_*xx*_), both transmitting and receiving antennas were placed co-polarized, either vertical (for *T*_*yy*_) or horizontal (for *T*_*xx*_). To measure cross-polarized transmission, the two antennas were placed orthogonal to each other, i.e., the transmitting antenna was vertically while the receiving antenna was placed horizontally. For the measurements of the transmission components, the free space method was used through the following expression^38^:

7 $${S}_{21}^{sample callibration}= \frac{{S}_{21}^{sample}-{ S}_{21}^{metal}}{{S}_{21}^{air} -{ S}_{21}^{metal}} {e}^{-j(w/c)d}$$

where *w*, c and d represent the angular frequency, speed of light and thickness of the sample, respectively. Figure 8c,d show that the simulated and experimental results are in good agreement. The measured phase difference between co- and cross-components remains within 90° ± 10° in all operating bands for CPS. Small discrepancies between simulated and measured results may be attributed to cable losses, calibration discontinuities, slight misplacement of antennas, and finite size of fabricated sample^39^.

Table 1 compares the performance of the proposed circular polarization selective metasurface with some previously reported bi-layered or tri-layered structures in terms of maximum polarization extinction ratio, angular stability, number of bands, number of layers and thickness. Improvement can be clearly seen in terms of PER, angular stability and number of bands but the proposed CPS metasurface has a larger thickness compared to other reported work listed in Table 1.

**Table 1:** Comparison of different CPS metasurfaces.

References	Maximum PER (dB)	Angular stability	No. of bands	No. of layers	Thickness (mm)
^26^	− 30.1	No	2	2	0.008
^28^	30.1	No	4	3	1.1
^27^	21.45	No	3	3	1.2
^29^	< 10	No	1	2	1.5
^30^	− 21.1	No	2	2	1.5
^31^	20.74	No	2	2	1.5
^32^	37.3	No	1	2	1.524
This work	47.16	Up to 45°	4	2	1.57

## Conclusion

This paper presents a chiral metasurface which performs multiband asymmetric linear-to-circular polarization. The CPS metasurface design has an array of split ring resonators with a cross-shaped structure inside it. It is shown that the bi-layered metasurface has the ability to achieve circular polarization selectivity at frequency band between 5.18 and 5.23 GHz for a normally incident TE polarized wave and also achieve CPS operation at different range of frequencies of 10.64–10.82 GHz, 12.25–12.47 GHz and 14.42–14.67 GHz for a normally incident TM polarized wave. For 5.18–5.23 GHz frequency band, the transmission goes up to − 4 dB at 5.2 GHz while for the frequency band of 14.42–14.67 GHz, PER goes up to 47.16 dB at 14.53 GHz, which are the maximum values that has been achieved till to date to the best of authors’ knowledge. Owing to the miniaturization in unit cell size, polarization selectivity and angular stability up to 45°, the designed metasurface can be suitable for polarization conversion and biomedical applications.

## Data Availability

The datasets generated during and/or analyzed during the current study are available from the corresponding author on reasonable request.
